# Supplementary material for: Effect of Non-Genetic Factors on Reproduction of Extensive versus Intensive Florida Dairy Goats
Source: Vet Sci. 2022 Apr 30;9(5):219. doi: 10.3390/vetsci9050219 (PMC9142956; doi:10.3390/vetsci9050219)
Supplement: Supplementary file 1 [file vetsci-09-00219-s001.zip › vetsci-1681131-supplementary.pdf]

veterinary sciences

MDPI

Supplementary material

Table S1. Effect tests of the Generalized Linear Model (GLM) for age at first kidding in primiparous Florida goats.

Source	DF	L-R ChiSquare	Prob>ChiSq
Production system	1	89.857732	< 0.0001
Birth year	1	220.3907	< 0.0001
Birth season	3	239.20159	< 0.0001
Production system x Birth year	1	11.17099	0.0008
Production system x Birth season	3	109.75964	< 0.0001
Birth season x Birth year	3	37.665144	< 0.0001
Production system x Birth year x Birth season	3	29.225186	< 0.0001

Whole model: DF: 15; L-R ChiSquare: 1016.915; Prob > ChiSq: $p < 0.001$.

Table S2. Effect tests of the Generalized Linear Model (GLM) for kidding interval in multiparous Florida goats.

Source	DF	L-R ChiSquare	Prob>ChiSq
Production system	1	0.398548	0.5278
Lactation number	4	54.376501	< 0.0001*
Type of kidding	2	9.474654	0.0088*
Kidding year	1	1.4491605	0.2287
Kidding season	3	19.519195	0.0002*
Dry period of the previous lactation	3	539.15701	< 0.0001*
Production system x Lactation number	4	3.4593634	0.4841
Production system x Type of kidding	2	8.6050862	0.0135*
Production system x Kidding year	1	1.6084657	0.2047
Production system x Kidding season	3	2.480894	0.4788
Production system x Dry period of the previous lactation	3	0.3961241	0.9410
Lactation number x Type of kidding	8	38.91693	< 0.001*
Lactation number x Kidding year	4	32.742038	< 0.001*
Lactation number x Kidding season	12	141.4737	< 0.001*
Lactation number x Dry period of the previous lactation	12	93.148295	< 0.001*
Type of kidding x Kidding year	2	13.516223	0.0012*
Type of kidding x Kidding season	6	12.94961	0.0438*
Type of kidding x Dry period of the previous lactation	6	2.9835178	0.8109
Kidding year x Kidding season	3	21.546444	< 0.0001*
Kidding year x Dry period of the previous lactation	3	4.5406746	0,2087
Kidding season x Dry period of the previous lactation	9	102.30823	<,0001*
Production system x Lactation number x Type of kidding	8	19.195645	0.0138*
Production system x Lactation number x Kidding year	4	8.6644736	0.0701
Production system x Lactation number x Kidding season	12	76.481317	< 0.0001*
Production system x Lactation number x Dry period of the previous lactation	12	18.69577	0.0961
Production system x Type of kidding x Kidding year	2	0.0609936	0.9700
Production system x Type of kidding x Kidding season	6	18.023035	0.0062*
Production system x Type of kidding x Dry period of the previous lactation	6	7.6010206	0.2688
Production system x Kidding year x Kidding season	3	15.66609	0.0013*
Production system x Kidding year x Dry period of the previous lactation	3	9.8160492	0.0202*
Production system x Kidding season x Dry period of the previous lactation	9	69.457325	< 0.0001*
Lactation number x Type of kidding x Kidding year	8	12.587115	0.1269
Lactation number x Type of kidding x Kidding season	24	34.689003	0.0731
Lactation number x Type of kidding x Dry period of the previous lactation	24	62.85053	< 0.0001*
Lactation number x Kidding year x Kidding season	12	43.62672	< 0.0001*
Lactation number x Kidding year x Dry period of the previous lactation	12	48.949589	< 0.0001*
Lactation number x Kidding season x Dry period of the previous lactation	36	194.61237	< 0.0001*
Type of kidding x Kidding year x Kidding season	6	20.25101	0.0025*
Type of kidding x Kidding year x Dry period of the previous lactation	6	34.282418	< 0.0001*
Type of kidding x Kidding season x Dry period of the previous lactation	18	80.094843	< 0.0001*
Kidding year x Kidding season x Dry period of the previous lactation	9	54.078894	< 0.0001*

Whole model: DF: 312; L-R ChiSquare: 10137.58; Prob > ChiSq: $p < 0.001$. *: significative results

Table S3. Effect tests of the Generalized Linear Model (GLM) for prolificacy in primiparous Florida goats.

Source	DF	L-R ChiSquare	Prob>ChiSq
Production system	1	17.976557	< 0.0001*
Kidding year	1	89.462834	< 0.0001*
Kidding season	3	5.8290473	0.1202
Age at First Kidding	2	21.394988	< 0.0001*
Production system x Age at First Kidding	2	0.3866391	0.8242
Production system x Kidding year	1	1.2618343	0.2613
Production system x Kidding season	3	31.549138	< 0.0001*
Age at First Kidding x Kidding year-cat	2	7.4230595	0.0244*
Age at First Kidding x Kidding season	6	14.410496	0.0254*
Kidding year x Kidding season	3	8.4004231	0.0384*
Production system x Age at First Kidding x Kidding year	2	1.6689346	0.4341
Production system x Age at First Kidding x Kidding season	6	13.747749	0.0326*
Production system x Kidding year x Kidding season	3	9.1257106	0.0277*
Age at First Kidding x Kidding year x Kidding season	6	64.347244	< 0.0001*

Whole model: DF: 41; L-R ChiSquare: 1145.557; Prob > ChiSq: $p < 0.001$. *: significative results

Table S4. Effect tests of the Generalized Linear Model (GLM) for prolificacy in multiparous Florida goats.

Source	DF	L-R ChiSquare	Prob>ChiSq
Production system	1	7.4582508	0.0063*
Kidding year	1	67.262519	< 0.0001*
Kidding season	3	23.604416	< 0.0001*
Lactation number	4	58.362998	< 0.0001*
Kidding interval	2	21.55524	< 0.0001*
Production system x Lactation number	4	24.930867	< 0.0001*
Production system x Kidding interval	2	1.4413976	0.4864
Production system x Kidding year	1	67.90127	< 0.0001*
Production system x Kidding season	3	6.3905675	0.0941
Lactation number x Kidding interval	8	10.128141	0.2561
Lactation number x Kidding year	4	16.324891	0.0026*
Lactation number x Kidding season	12	24.111894	0.0196*
Kidding interval x Kidding year	2	3.7731312	0.1516
Kidding interval x Kidding season	6	11.890335	0.0645
Kidding year x Kidding season	3	20.657707	0.0001*
Production system x Lactation number x Kidding interval	8	22.675499	0.0038*
Production system x Lactation number x Kidding year	4	21.92997	0.0002*
Production system x Lactation number x Kidding season	12	40.372304	< 0.0001*
Production system x Kidding interval x Kidding year	2	0.6986553	0.7052
Production system x Kidding interval x Kidding season	6	17.687084	0.0071*
Production system x Kidding year x Kidding season	3	9.0533756	0.0286*
Lactation number x Kidding interval x Kidding year	8	20.102375	0.0100*
Lactation number x Kidding interval x Kidding season	24	58.834424	< 0.0001*
Lactation number x Kidding year x Kidding season	12	23.492013	0.0238*
Kidding interval x Kidding year x Kidding season	6	37.258553	< 0.0001*

Whole model: DF: 141; L-R ChiSquare: 1156.009; Prob > ChiSq: $p < 0.001$. *: significative results
